# NUDT21 interacts with NDUFS2 to activate the PI3K/AKT pathway and promotes pancreatic cancer pathogenesis

**DOI:** 10.1007/s00432-023-05540-1

**Published:** 2024-01-09

**Authors:** Xiao-Dong Huang, Yong-Wei Chen, Lv Tian, Li Du, Xiao-Chen Cheng, Yu-Xin Lu, Dong-Dong Lin, Feng-Jun Xiao

**Affiliations:** 1 Department of General Surgery, Xuanwu Hospital Capital Medical University, Beijing, 100053 People’s Republic of China; 2 Faculty of Hepato-Pancreato-Biliary Surgery, The First Medical Center of PLA General Hospital, Beijing, 100853 People’s Republic of China; 3 School of Nursing, Jilin University, Changchun, 130015 People’s Republic of China; 4 Department of Experimental Hematology and Biochemistry, Beijing Institute of Radiation Medicine, Beijing, 100850 People’s Republic of China

**Keywords:** Pancreatic adenocarcinoma, NUDT21, NDUFS2, PI3K, AKT pathway

## Abstract

**Background:**

NUDT21 (Nudix Hydrolase 21) has been shown to play an essential role in multiple biological processes. Pancreatic adenocarcinoma (PAAD) is one of the most fatal cancers in the world. However, the biological function of NUDT21 in PAAD remains rarely understood. The aim of this research was to identify the prediction value of NUDT21 in diagnosis, prognosis, immune infiltration, and signal pathway in PAAD.

**Methods:**

Combined with the data in online databases, we analyzed the expression, immune infiltration, function enrichment, signal pathway, diagnosis, and prognosis of NUDT21 in PAAD. Then, the biological function of NUDT21 and its interacted protein in PAAD was identified through plasmid transduction system and protein mass spectrometry. Expression of NUDT21 was further verified in clinical specimens by immunofluorescence.

**Results:**

We found that NUDT21 was upregulated in PAAD tissues and was significantly associated with the diagnosis and prognosis of pancreatic cancer through bioinformatic data analysis. We also found that overexpression of NUDT21 enhanced PAAD cells proliferation and migration, whereas knockdown NUDT21 restored the effects through in vitro experiment. Moreover, NDUFS2 was recognized as a potential target of NUDT21.We further verified that the expression of NDUFS2 was positively correlated with NUDT21 in PAAD clinical specimens. Mechanically, we found that NUDT21 stabilizes NDUFS2 and activates the PI3K-AKT signaling pathway.

**Conclusion:**

Our investigation reveals that NUDT21 is a previously unrecognized oncogenic factor in the diagnosis, prognosis, and treatment target of PAAD, and we suggest that NUDT21 might be a novel therapeutic target in PAAD.

**Supplementary Information:**

The online version contains supplementary material available at 10.1007/s00432-023-05540-1.

## Introduction

Pancreatic cancer is the fourth leading cause of all cancer-related deaths, and it has an overall 5-year survival rate of less than 10% due to late diagnosis, frequent metastases, limited treatment options, and poor prognosis (Lee et al. 2021; Jia et al. 2019). Only a minority of patients are eligible for surgery as the disease has often progressed locally or metastasized distantly at the time of diagnosis (Li et al. 2004). In recent years, with the increasing popularity of minimally invasive techniques and adjuvant surgical treatments, the quality and safety of pancreatic cancer surgery have improved. Chemotherapy is now increasingly used as neoadjuvant therapy and is usually accompanied by radiotherapy in PAAD (Kolbeinsson et al. 2023). Extensive testing of the pancreatic cancer genome has also led to a better understanding of the biology of pancreatic cancer, and targeted therapies and immunotherapies have brought new approaches to the treatment of pancreatic cancer (Dalmasso et al. 2022; Qiu et al. 2023). Unfortunately, to date, the efficacy of immune checkpoint inhibitors in patients with pancreatic cancer is not satisfactory (Mukherji et al. 2022). In general, pancreatic cancer is still a disease with a low long-term survival rate, and there is still an urgent need to find new therapeutic targets and treatment methods.

NUDT21 is a key mediator involved in alternative polyadenylation (APA) and pre-mRNA 3′ end processing (Ruegsegger et al. 1998). NUDT21 functions by binding to the UGUA sequence of pre-mRNA (Masamha 2023) and plays a broader role in cell fate changes and chromatin signaling (Brumbaugh et al. 2018). NUDT21 has emerged as an important regulator of cell fate decisions in normal and pathological conditions, but its involvement in cancer is not fully understood (Yang et al. 2020; Abadi et al. 2019). Decreased NUDT21 expression has been reported to promote cell proliferation in glioblastoma, bladder cancer, and hepatocellular carcinoma (Xiong et al. 2019; Masamha et al. 2014; Han and Kim 2014; Tan et al. 2018), and knockdown of NUDT21 also inhibits the proliferation and promotes apoptosis of human K562 leukemia cells through the ERK pathway (Zhang and Zhang 2018); NUDT21 also promotes the proliferation and metastasis in human gastric cancer through modulating SGPP2 (Zhu et al. 2021). The available literature provides evidence supporting the notion that NUDT21 plays a dual role (both oncogenic and tumor suppressor) in the tumorigenesis of different tissues. However, the role and regulation pathway of NUDT21 in PAAD have rarely been reported, and our study will explore the function and possible regulation pathway of NUDT21 in PAAD through bioinformatic data research and in *vitro* experiments.

In this study, we will explore the expression of NUDT21 in pancreatic cancer and its effect on patients’ diagnosis and prognosis through preliminary bioinformatic data research, and analyze whether NUDT21 is an oncogene in pancreatic cancer. The effects of NUDT21 on the biological effects of pancreatic cancer cells will be explored by knockdown and overexpression of NUDT21 in pancreatic cancer cell lines. Protein mass spectrometry analysis was performed to identify possible interacted proteins with NUDT21 and possible downstream signaling pathways. As a regulator of RNA processing events, we will explore whether NUDT21 could provide a new feasible therapeutic target for the diagnosis and treatment of pancreatic cancer.

## Materials and methods

### Data sources and processing

Differential expression data of NUDT21 in PAAD and pan-cancer were obtained from UCSC XENA https://xenabrowser.net/datapages/ in the TPM format of the TCGA and GTEx processed uniformly by the Toil process (Vivian et al. 2017). Differential RNAseq expression of NUDT21 data were extracted in level 3 HTSeq‐FPKM format from the TCGA (https://portal.gdc.cancer.gov/ PAAD project. The differential expression of NUDT21 in PAAD at the transcriptome level were downloaded from the TCGA and GTEx online database http://gepia.cancer-pku.cn/. RNAseq data in FPKM (Fragments per Kilobase per Million) format were converted to TPM (Transcripts per Million Reads) format and log transformed. All final analyses were performed using data in TPM format. All statistical analyses and visualizations were performed using R (version 4.2.1).

### Immune infiltration analysis of NUDT21

We selected 24 immune cell markers (Bindea et al. 2013) and analyzed the relative infiltration levels of 24 immune cells in PAAD using the GSVA software package (version 24.1.34) (Hanzelmann et al. 2013). The immune infiltration algorithm was ssGSEA and correlation analysis was performed using Spearman. The samples were divided into low and high expression groups based on the expression of NUDT21, and the enrichment scores of various immune cell infiltrates in the different expression groups were calculated and analyzed using the GSVA software package (version 1.34.0). Correlations between NUDT21 and the expression of immune checkpoint-programmed cell death protein 1 (PD-1)-PDCD1 and cytotoxic T-lymphocyte-associated protein 4 (CTLA4) were assessed using Spearman analysis. We validated the immune infiltration of NUDT21 in PAAD using the online bioinformatics analysis tool TIMER2 (http://timer.cistrome.org/). Finally, immune cells with statistically significant relative infiltration (*p* < 0.001) were analyzed. The visualization uses the circlize package (version 0.4.12).

### Functional enrichment analysis of NUDT21 in PAAD

The results of single-gene differential analysis were analyzed by GO, KEGG, and GSEA functional enrichment using the clusterProfiler package (version 3.14.3) (Yu et al. 2012). Gene ID conversion was performed using the org.Hs.eg.db package (version 3.10.0) and the correlation of NUDT21 with the enriched pathways was scored by calculating Z values using the GOplot package (version 1.0.2) (Walter et al. 2015) which scores the relevance of NUDT21 to the enrichment pathway. The reference gene set used for the GSEA was c2.cgp. v7.2.symbols.gmt (Curated) (Subramanian et al. 2005) and the results were significantly enriched if they met the conditions of false discovery rate (FDR) < 0.25 and *p*.adjust < 0.05.

### Differential expression analysis of NUDT21

The Mann–Whitney U test (Wilcoxon rank sum test) was used to analyze the differences in the expression of NUDT21 in different cancers. Shapiro–Wilk normality test was used to test the normality of the data on the expression of NUDT21 in the unpaired samples. The independent samples t test was used to analyze the differences in the data in unpaired samples. Volcano plots were drawn using the results of the gene difference analyses with a threshold set at |log_2_(FC)|> 1. Volcano map was generated by ggplot2 (version 3.3.3). We also investigated the differential expression of NUDT21and NDUFS2 in PAAD using an online bioinformatics analysis tool GEPIA2 (http://gepia2.cancer-pku.cn/). We also validated the differential expression of NUDT21 in pan-cancer using an online bioinformatics analysis tool TIMER2 (http://timer.cistrome.org/). All the above analyses were considered statistically significant when *p* < 0.05.

### Clinical correlation analysis, survival prognosis analysis, and construction of prognostic models

We statistically analyzed the survival data of PAAD patients using the survival software package (version 3.2–10) and plotted the overall survival (OS) and Kaplan–Meier survival curves of PAAD patients using the survminer software package (version 0.4.9). We then analyzed the Kaplan–Meier survival curves of PAAD patients in subgroups according to clinicopathological factors such as age and gender. We then used these clinicopathological factors to calculate their correlation with NUDT21 expression and visualized the results of the calculations using ggplot2 (version 3.3.3). We performed ROC analysis of the data using the pROC software package (version 1.17.0.1) to determine the accuracy of NUDT21 in predicting prognosis.

### Cell lines and culture

The human pancreatic cancer PANC05.04 cells were cultured in F12k media (#21,127,022, Gibco) supplemented with 10% FBS and 100 × Penicillin–Streptomycin Solution. Cells were incubated in a humidified incubator at 37 °C with 5% CO_2_.

### Cell transfection

sh-NUDT21, OE-NUDT21, sh-NDUFS2, and their each control plasmids were purchased from Genechem (Shanghai, China) company. Transient transfections were performed with jetPRIME (#150–15, Polyplus, France) transfection reagent following the manufacturer’s instructions.

### Cell proliferation assay

Cell viability was quantified using the Cell Counting Kit-8 assay (B34304, Selleckchem). Cells were seeded into 96-well plates at a density of 3 × 10^3 ^cells/well and were cultured in a humidified atmosphere with 5% CO_2_ at 37 °C. Subsequently, 10 μl CCK-8 reagent was added to each well. The absorbance was measured at 450 nm after 2 h. The CCK-8 assay was performed every 24 h for 4 consecutive days.

### Cell apoptosis assay

The Annexin V-FITC/PI Apoptosis Detection Kit (Multi Sciences Biotech Co., Ltd.) was used in the present study. Briefly, cells were seeded into 6-well plates and were harvested and resuspended in 500 μl binding buffer after 48 h. Subsequently, cells were incubated with 5 μl Annexin V-FITC and 10 μl PI at room temperature in the dark for 15 min before analysis via flow cytometry.

### Colony formation assay

For colony formation analysis, 1000–1500 cells per well were plated in six-well plates and allowed to grow for 14 days after the indicated treatments, then we fixed the cells with 4% paraformaldehyde (P1110,Solarbio), and stained the cells with 5% crystal violet (G1063,Solarbio).

### Cell cycle assay

The Cell Cycle Staining Kit (CCS012, Multi Sciences Biotech Co., Ltd.) was used according to the manufacturer’s protocol. Briefly, cells were harvested and fixed in 70% ethanol at 4 °C overnight. Cells were then treated with 1 ml DNA staining solution and 10 μl permeabilization at 37 °C in the dark for 30 min. Cells were then analyzed via flow cytometry.

### Transwell migration assay

2 × 10^5^ cells which were suspended in 1.5 ml serum-free culture media were added to the top chamber of 6-well transwell plates (3428, Corning), and 2.6 ml culture media containing 10% FBS were added to the bottom chamber. After incubating at 37 °C for 48 h, the chambers were washed with PBS twice, and these cells which migrated to the bottom chambers were fixed with 4% paraformaldehyde and stained with crystal violet. Then the number of transitional cells in all chambers was calculated in the 5 visual fields.

### Wound healing assay

Cells were seeded on 6-well plate and grew to the pavement overnight. After 24 h of transfection, a channel was drawn on the monolayer cells with 10 μl micropipette tip. Then cells were washed with PBS twice and cultured in serum-free culture media at 5% CO_2_, 37 °C for an additional 24 h. Photographs were taken by an inverted Leica phase contrast microscope at 0 h and 24 h.

### NADP^+^/NADPH and ATP assay

For measurements of NADP + /NADPH ratio and ATP concentration, cells were harvested and treated with appropriate buffers as indicated by the providers. In particular, NADP + /NADPH ratios were determined using a NADP + /NADPH Assay kit (S0179, Beyotime) and were detected using a colorimetric assay under a microplate reader (Feyond-A400, ALLSHENG, China) with detection wavelengths of 570 nm, respectively. The ATP concentration was calculated using an enhanced ATP assay kit (S0027, Beyotime) by measuring chemiluminescence with a luminometer plate reader.

### Western blotting

Cells were lysed using RIPA buffer (P0013B, Beyotime Biotechnology). Subsequently, total protein was separated using SDS-PAGE and transferred to a PVDF membrane. Membranes were blocked and were subsequently incubated at 4 °C overnight with the following primary antibodies against: GAPDH (2118, 1:1000; CST.), beta-Tubulin (16,305, 1:2000; CST), NDUFS2 (sc-390596, 1:1000; MDL Biological, Inc.), AKT (ab8805, 1:1000; Abcam), P-AKT308(ab38449, 1:1000; Abcam), NUDT21 (ab183660, 1/1000; Abcam), PI3K(#4257, 1/1000; CST), and P-PI3K(#4228, 1/1000; CST). All antibodies were used according to the manufacturer’s protocol. Following primary incubation, membranes were incubated with secondary antibodies, HRP-conjugated goat anti-rabbit IgG (PR30011, 1:20,000; Proteintech Co., Ltd.) and HRP-conjugated goat anti-mouse IgG (PR30012, 1:20,000; Proteintech Co., Ltd.) at room temperature for 1 h. Proteins were visualized using ECL Reagent (42,029,053, Millipore Sigma).

### Immunofluorescence staining and co-immunoprecipitation

Slides containing human pancreatic tumor tissues or cells were used in our experiment. The slides were incubated with primary antibodies at 4 °C overnight. The following day, after incubation with the corresponding secondary antibody, the nuclei were stained with DAPI (Yeason, 40728ES03) before observing by confocal laser microscopy. Co-immunoprecipitation analysis was carried out using the Pierce Co-Immunoprecipitation Kit (88,804; Thermo Fisher) according to the manufacturer’s instructions.

### Drugs and inhibitors

100 μM insulin which is a P-AKT activator and 1 μM wortmannin (CST, #9951) which is an inhibitor of PI3 kinase were used to treat pancreatic cell lines.

### Clinical samples

The protocol of this retrospective study was approved by the Ethics Committee of the PLA General Hospital, and all enrolled patients were informed of and agreed to participate in this study by signing a written informed consent form. In this study, paraffin-embedded specimens from 30 patients diagnosed with PAAD between September 1, 2018 and June 1, 2019 were collected from the Department of Hepatobiliary Surgery of the PLA General Hospital, and the baseline characteristics and pathological data of the patients were obtained from the database of the PLA General Hospital.

### Statistical analysis

Data are expressed as mean ± standard deviation (mean ± SD). Differences in the expression of NUDT21 in PAAD tumor tissues and adjacent tissues were analyzed by Student’s t test. Comparisons between multiple groups were performed using one-way analysis of variance (ANOVA). The correlation between NUDT21 expression and clinical data of PAAD patients was analyzed using Mann–Whitney U test. Statistical graphs were completed using GraphPad Prism 9, and P < 0.05 was considered a statistically significant difference.

## Results

### Functional enrichment analysis of NUDT21 in PAAD

**Fig. 1:**
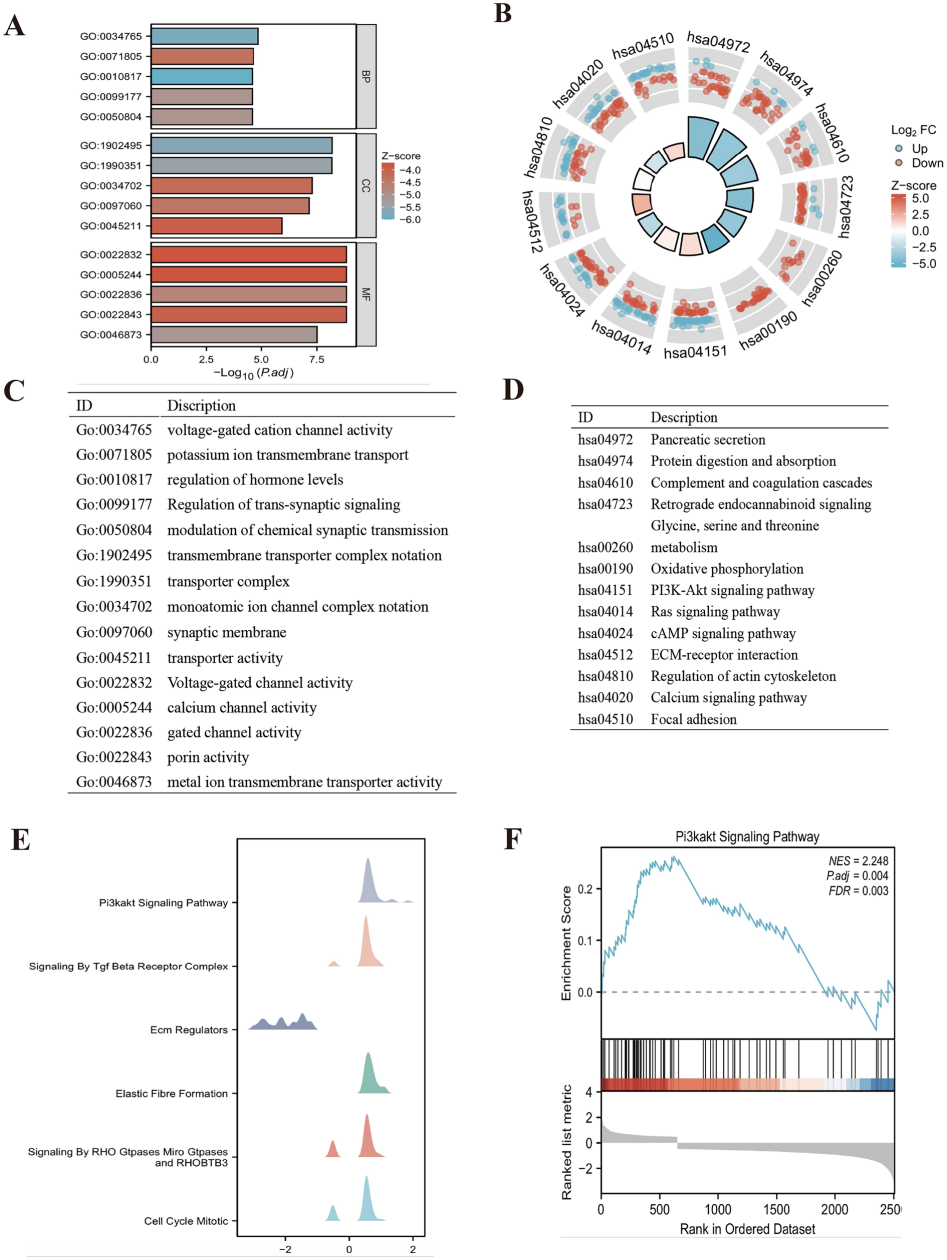
Functional enrichment analysis of NUDT21 in PAAD. **A** Results of GO analysis. **B** Results of KEGG analysis. **C**, **D** GO and KEGG analysis category names corresponding to GO and KEGG identifiers. **E**, **F** The results of GSEA. When the horizontal coordinate is positive, it suggests that the expression of NUDT21 is positively correlated with the pathway, while the opposite is true when the horizontal coordinate is negative

GO, KEGG, and GSEA enrichment analyses were performed using the results of single-gene differential analysis, and the results are shown in Fig. 1. Figure 1A, C and Table 1 show the results of GO analysis, which revealed that NUDT21 is functionally related to voltage-gated channel activity, calcium channel activity, gated channel activity, and porin activity. Figure 1B, D and Table 1 show the results of KEGG analysis, which revealed that NUDT21 was associated with PI3K-AKT signaling pathway, ECM–receptor interaction, focal adhesion, etc. The Z score reflects the correlation of NUDT21 with these pathways to some extent. A negative Z score indicates a negative correlation, and a positive Z score indicates a positive correlation. Figure 1E, F shows the enrichment and grading results of GSEA, which suggested that there was significant enrichment in PI3K-Akt signaling pathway, signaling by Tgf beta receptor complex, elastic fiber formation, etc., suggesting that NUDT21 was indeed closely related to cancer.

**Table 1:** GO and KEGG analysis

Ontology	ID	Description	GeneRatio	BgRatio	*P* value	p.adjust	Q value
BP	GO:0034765	Regulation of ion transmembrane transport	100/2190	476/18800	2.4254E-09	1.4223E-05	1.3E-05
BP	GO:0071805	Potassium ion transmembrane transport	56/2190	219/18800	8.0199E-09	2.3514E-05	2.1493E-05
BP	GO:0050804	Modulation of chemical synaptic transmission	90/2190	429/18800	1.6366E-08	2.6422E-05	2.4151E-05
BP	GO:0099177	Regulation of trans-synaptic signaling	90/2190	430/18800	1.8382E-08	2.6422E-05	2.4151E-05
BP	GO:0010817	Regulation of hormone levels	100/2190	496/18800	2.2529E-08	2.6422E-05	2.4151E-05
CC	GO:1,902,495	Transmembrane transporter complex	92/2337	377/19594	9.4126E-12	6.5229E-09	5.6476E-09
CC	GO:1,990,351	Transporter complex	95/2337	399/19594	1.9141E-11	6.6325E-09	5.7424E-09
CC	GO:0034702	Ion channel complex	74/2337	294/19594	2.2403E-10	5.1751E-08	4.4806E-08
CC	GO:0097060	Synaptic membrane	87/2337	373/19594	4.1102E-10	7.1209E-08	6.1653E-08
CC	GO:0045211	Postsynaptic membrane	66/2337	271/19594	8.6651E-09	1.201E-06	1.0398E-06
MF	GO:0022843	Voltage-gated cation channel activity	50/2245	144/18410	1.7206E-12	1.4766E-09	1.3162E-09
MF	GO:0022836	Gated channel activity	88/2245	340/18410	2.8168E-12	1.4766E-09	1.3162E-09
MF	GO:0005244	Voltage-gated ion channel activity	61/2245	201/18410	5.3548E-12	1.4766E-09	1.3162E-09
MF	GO:0022832	Voltage-gated channel activity	61/2245	201/18410	5.3548E-12	1.4766E-09	1.3162E-09
MF	GO:0046873	Metal ion transmembrane transporter activity	99/2245	428/18410	1.4273E-10	3.1358E-08	2.7951E-08
KEGG	hsa04972	Pancreatic secretion	32/956	102/8164	8.5127E-08	1.3961E-05	1.1963E-05
KEGG	hsa04974	Protein digestion and absorption	31/956	103/8164	3.8355E-07	4.1935E-05	3.5933E-05
KEGG	hsa04610	Complement and coagulation cascades	26/956	85/8164	2.363E-06	0.00019377	0.00016604
KEGG	hsa04723	Retrograde endocannabinoid signaling	35/956	148/8164	3.0067E-05	0.00140884	0.00120719
KEGG	hsa00260	Glycine, serine and threonine metabolism	14/956	40/8164	0.00010189	0.00352101	0.00301704
KEGG	hsa00190	Oxidative phosphorylation	31/956	134/8164	0.00013051	0.00385408	0.00330244
KEGG	hsa04151	PI3K-Akt signaling pathway	64/956	354/8164	0.00021557	0.00543892	0.00466044
KEGG	hsa04014	Ras signaling pathway	45/956	235/8164	0.00052425	0.01146359	0.00982278
KEGG	hsa04024	cAMP signaling pathway	42/956	221/8164	0.00093094	0.01908422	0.01635265
KEGG	hsa04512	ECM–receptor interaction	21/956	88/8164	0.00099937	0.01928201	0.01652213
KEGG	hsa04810	Regulation of actin cytoskeleton	41/956	218/8164	0.00131473	0.02227862	0.01908983
KEGG	hsa04020	Calcium signaling pathway	43/956	240/8164	0.0026987	0.04215109	0.03611791
KEGG	hsa04510	Focal adhesion	37/956	201/8164	0.00324491	0.04837861	0.04145407

### Immune infiltration, prognostic analysis, and clinical correlation analysis of NUDT21 in PAAD

**Fig. 2:**
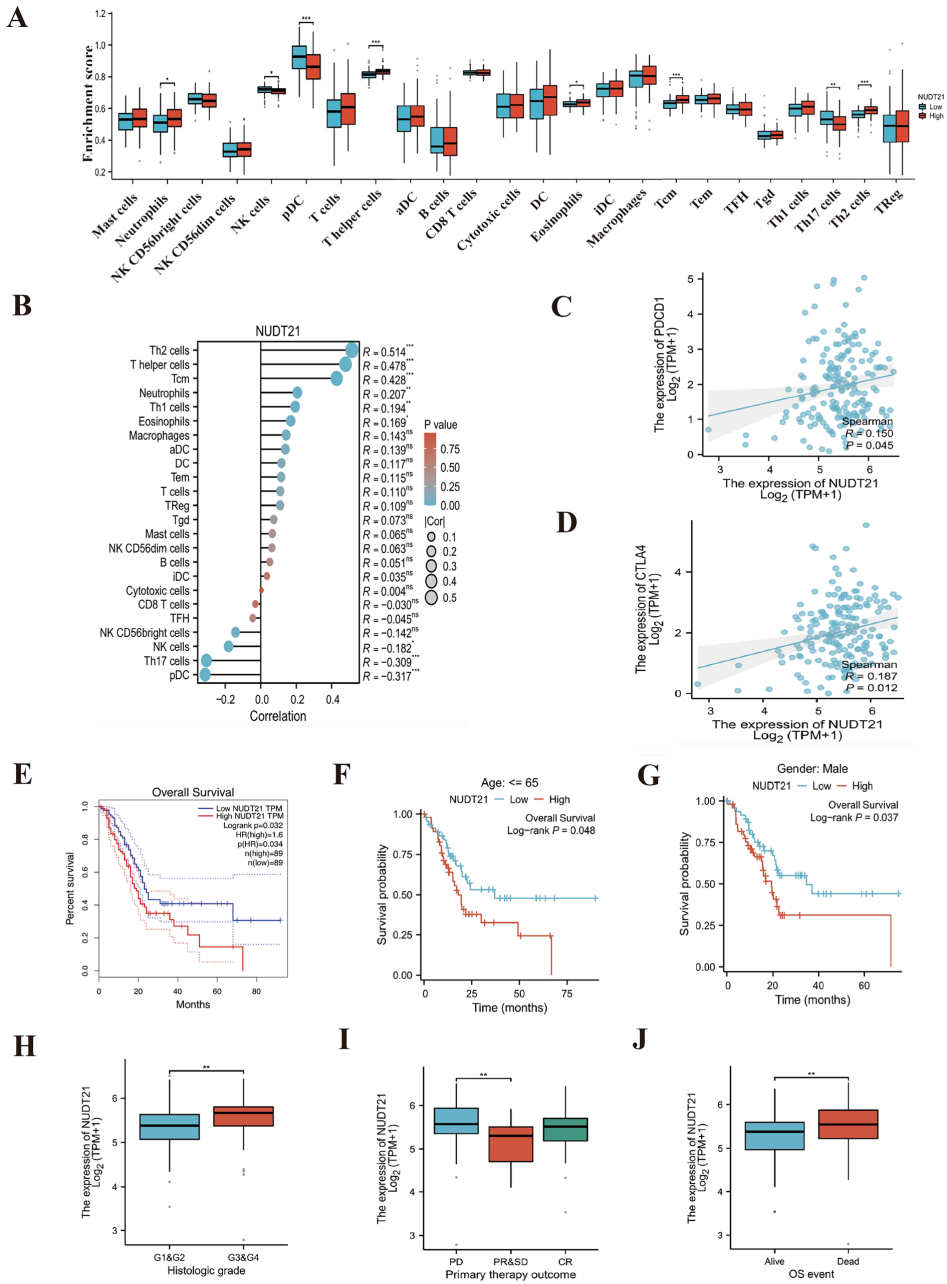
Immune infiltration and prognostic analysis of NUDT21. **A** Grouped comparison of the infiltration levels of 24 immune cells in the low and high expression groups of NUDT21. **B** Results of ssGSEA of the correlation between the expression of NUDT21 and 24 immune cells. **C** The correlation between the expression of NUDT21 and PDCD1. **D** The correlation between the expression of NUDT21 and CTLA4. **E** Kaplan–Meier survival curves of the relationship between NUDT21 expression and overall survival (OS) in PAAD. **F**, **G** Kaplan–Meier survival curves of the relationship between NUDT21 expression and OS in different patient subgroups with distinct clinicopathological factors. **H**–**J** Expression levels of NUDT21 in different groups of patients with distinct clinicopathological factors

To determine the effect of NUDT21 expression on the tumor microenvironment, immune infiltration analysis was performed using the ssGSEA method. The correlation between immune cell enrichment and NUDT21 expression levels in pancreatic tissues was calculated using Spearman correlation analysis. The results are shown in Fig. 2A.The expression of NUDT21 was positively correlated with the level of infiltration of five immune cells, namely, Neutrophils, T helper cells, Eosinophils, and Th2 cells. The expression of NUDT21 was negatively correlated with the level of infiltration of three immune cells, namely, NK cells, pDC, and Th17 cells. Next, we divided the expression profile data into high and low expression groups according to the expression level of NUDT21 to identify the changes in the level of immune cell infiltration in different groups. The results, which are shown in Fig. 2B, indicate that in Th2 cells, T helper cells, Tcm Neutrophils, Th1 cells, and Eosinophils cells, the level of infiltration in the high expression group was significantly higher than that in the low expression group. In NK cells, Th17 cells, and pDC cells, the infiltration level of the low expression group was significantly higher than that of the high expression group, and this result is consistent with the results shown in Fig. 2A. We also searched the TCGA database for correlations between NUDT21 and PDCD1 (PD-1), CTLA4. We found that NUDT21 was positively correlated with the expression levels of PD-1 and CTLA4 (Fig. 2C, D).

Then, we divided the expression profile data into high and low expression groups according to the expression of NUDT21 and analyzed the overall survival (OS) of PAAD patients in the different groups. The results showed that the groups with high expression of NUDT21 exhibited shorter survival times (Fig. 2E). We then grouped PAAD patients according to clinicopathological factors and analyzed whether there would be a significant difference in OS between different subgroups. We found that among patients older than 65 years, with male gender, the group with high expression of NUDT21 showed shorter survival time (Fig. 2F, G). These results suggested that high expression of NUDT21 was associated with poor prognosis and was closely related to tumor development. Furthermore, these findings indicated that high expression of NUDT21 is a risk factor for patients of PAAD.

To further verify that high expression of NUDT21 is associated with poor prognosis, we compared the expression levels of NUDT21 in patients from different groups with distinct clinicopathological factors, and the results are shown in Fig. 2H–J. A clinical baseline information table was constructed based on the expression level of NUDT21, as shown in Table 2. The expression levels of NUDT21 in patients with different histological grades, primary therapy outcome, OS event, etc., were significantly different from those in control patients, which suggested that the expression of NUDT21 may be associated with tumorigenesis and can be used as a marker for tumor diagnosis.

**Table 2:** Clinical baseline information

Characteristics	Low expression of NUDT21(89)	High expression of NUDT21(90)	*P* value	Statistic
*Pathologic T st*age, *n* (%)			0.14229	5.43973
T1	3 (1.7%)	4 (2.3%)		
T2	15 (8.5%)	9 (5.1%)		
T3	66 (37.3%)	77 (43.5%)		
T4	3 (1.7%)	0 (0%)		
*Pathologic N stage*, *n* (%)			0.33730	0.92065
N0	27 (15.5%)	23 (13.2%)		
N1	57 (32.8%)	67 (38.5%)		
*Pathologic M stage*, *n* (%)			0.56447	0.33203
M0	35 (41.2%)	45 (52.9%)		
M1	1 (1.2%)	4 (4.7%)		
*Pathologic stage*, *n* (%)			0.14021	5.47382
Stage I	12 (6.8%)	9 (5.1%)		
Stage II	70 (39.8%)	77 (43.8%)		
Stage III	3 (1.7%)	0 (0%)		
Stage IV	1 (0.6%)	4 (2.3%)		
*Primary therapy outcome*, *n* (%)			0.44486	2.67284
PD	22 (15.7%)	28 (20%)		
SD	5 (3.6%)	4 (2.9%)		
PR	7 (5%)	3 (2.1%)		
CR	32 (22.9%)	39 (27.9%)		
*Gender*, *n* (%)			0.71297	0.13533
Female	41 (22.9%)	39 (21.8%)		
Male	48 (26.8%)	51 (28.5%)		
*Age*, *n* (%)			0.60299	0.27052
< = 65	45 (25.1%)	49 (27.4%)		
> 65	44 (24.6%)	41 (22.9%)		
*Residual tumor*, *n* (%)			0.29759	2.42406
R0	56 (33.9%)	51 (30.9%)		
R1	21 (12.7%)	32 (19.4%)		
R2	2 (1.2%)	3 (1.8%)		
*Histologic grade*, *n* (%)			0.01034	11.27294
G1	21 (11.9%)	10 (5.6%)		
G2	49 (27.7%)	47 (26.6%)		
G3	16 (9%)	32 (18.1%)		
G4	2 (1.1%)	0 (0%)		
*Anatomic neoplasm subdivision*, *n* (%)			0.25511	4.05953
Body	9 (5%)	5 (2.8%)		
Head	65 (36.3%)	74 (41.3%)		
Tail	7 (3.9%)	8 (4.5%)		
Other	8 (4.5%)	3 (1.7%)		
*Alcohol history*, *n* (%)			0.15547	2.01772
No	29 (17.4%)	36 (21.6%)		
Yes	57 (34.1%)	45 (26.9%)		
*History of diabetes*, *n* (%)			0.87339	0.02540
No	59 (40.1%)	50 (34%)		
Yes	20 (13.6%)	18 (12.2%)		
*History of chronic pancreatitis*, *n* (%)			0.19488	1.68031
No	65 (45.8%)	64 (45.1%)		
Yes	9 (6.3%)	4 (2.8%)		
*Family history of cancer*, *n* (%)			0.62062	0.24500
No	25 (22.5%)	22 (19.8%)		
Yes	31 (27.9%)	33 (29.7%)		
*Smoker*, *n* (%)			0.89193	0.01846
No	35 (24.1%)	31 (21.4%)		
Yes	41 (28.3%)	38 (26.2%)		
*Radiation therapy*, *n* (%)			0.43596	0.60689
No	58 (35.4%)	61 (37.2%)		
Yes	25 (15.2%)	20 (12.2%)		

### NUDT21 promotes proliferation and inhibits apoptosis of Panc05.04 cells

**Fig. 3:**
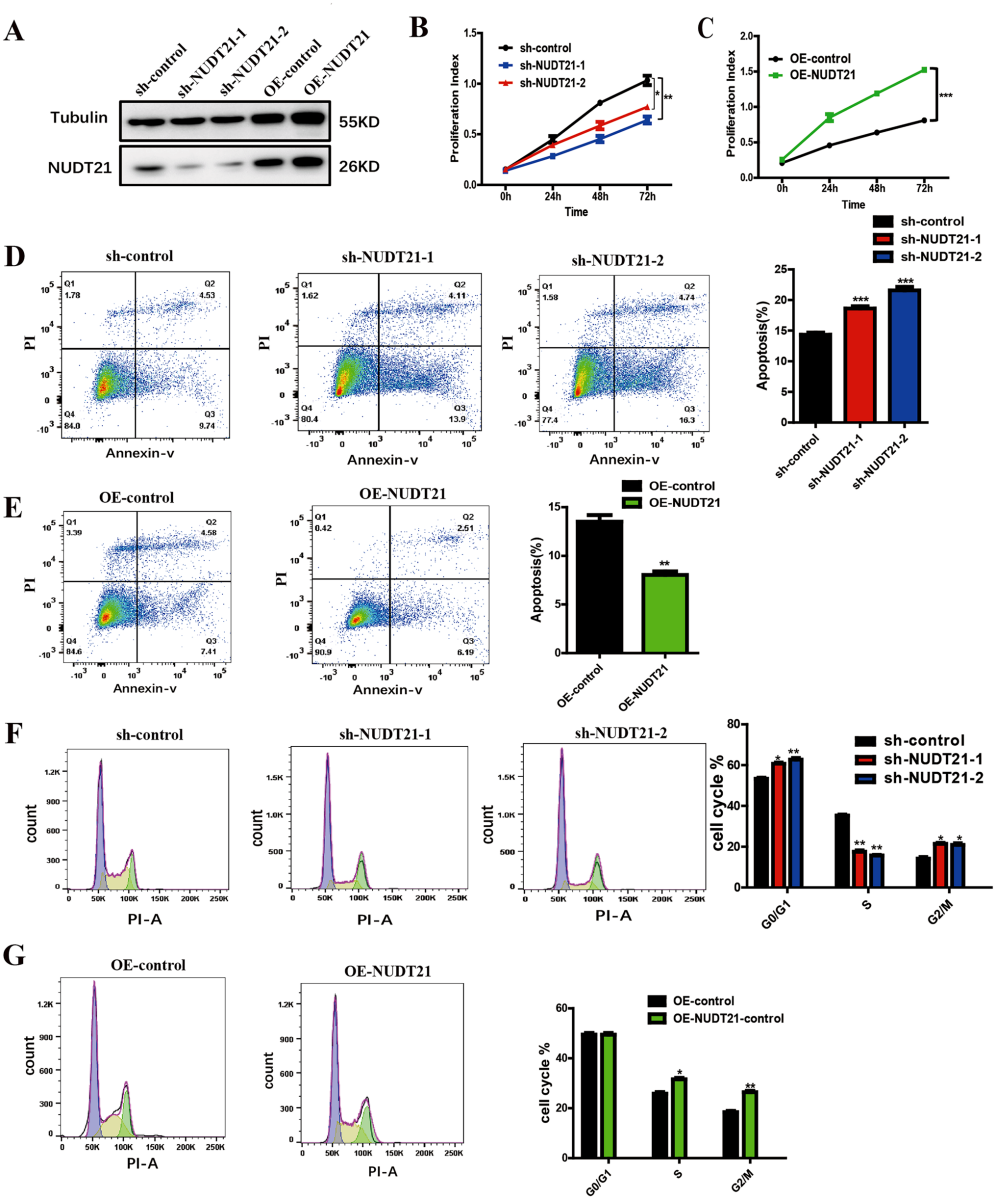
NUDT21 promotes the proliferation and suppresses apoptosis in pancreatic cancer cell lines. **A** Western blotting shows the expression of NDUFS2 in Panc05.04 cells transfected with sh-NUDT21 and OE-NUDT21 plasmid. **B**, **C** CCK-8 assay shows the proliferation index in Panc05.04 cells transfected with sh-NUDT21 and OE-NUDT21 plasmid. **D**, **E** Cell apoptosis assay shows the percentage of apoptotic cell in Panc05.04 cells transfected with sh-NUDT21 and OE-NUDT21 plasmid. **F**, **G** Cell cycle assay shows the percentage of cell cycle distribution of Panc05.04 cells transfected with sh-NUDT21 and OE-NUDT21 plasmid

To determine the biological function of NUDT21, knockdown and overexpression plasmids were used in our experiments. Western blotting showed the transduction rate of the knockdown and overexpression of NUDT21 plasmid (Fig. 3A). CCK8 assay was used to test the function of NUDT21 on the proliferation of pancreatic cancer cell line Panc05.04. We observed that interfering with the NUDT21 could significantly inhibit the proliferation of Panc05.04 cells, while overexpression of the NUDT21 gene could significantly promote the proliferation of PANC05.04 cells (Fig. 3B, C). Cell apoptosis assay was used to detect the changes in the number of apoptotic cells after knockdown and overexpression of NUDT21.The result shows that knockdown of NUDT21 promotes the apoptosis of Panc05.04 cells, while overexpression of NUDT21 suppresses the apoptosis of Panc05.04 cells (Fig. 3D, E). Flow cytometric was used to test the cell cycle, and the result showed that interfering with the NUDT21 gene could significantly suppress the S phase and prolong the G0/G1 and G2/M phases, while overexpression of the NUDT21 gene could significantly prolong the S phase and suppress the G0/G1 and G2/M phases (Fig. 3F, G).

### NUDT21 promotes migration and ATP production in Panc05.04 cells

**Fig. 4:**
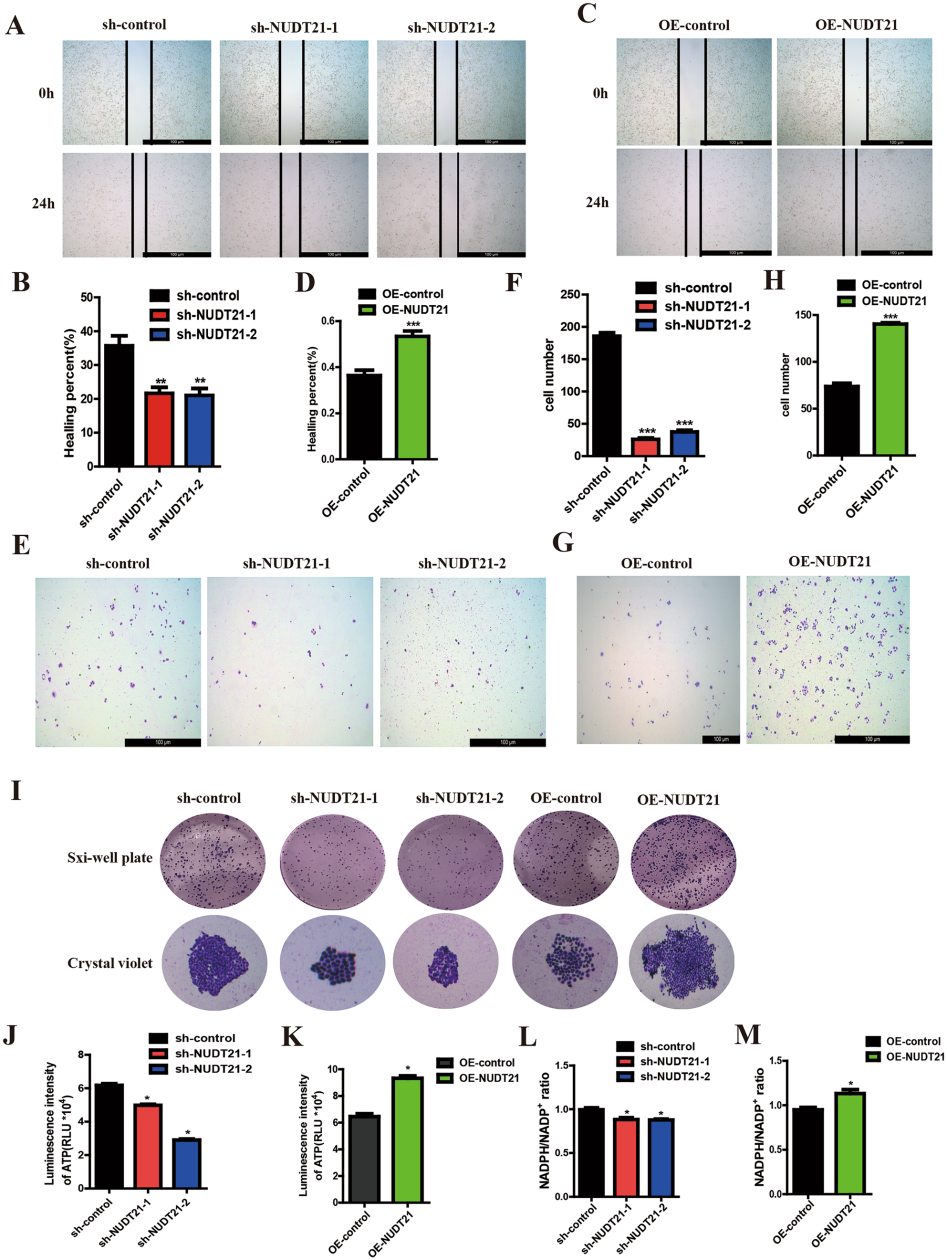
NUDT21 promotes the migration and ATP synthesis in pancreatic cancer cell lines **A**, **B** Wound healing assay shows the wound healing rate in Panc05.04 cells transfected with sh-NUDT21 plasmid. **C**, **D** Wound healing assay shows the wound healing rate in Panc05.04 cells transfected with OE-NUDT21 plasmid **E**, **F** Transwell migration assay shows the number of transitional cells that migrated to the bottom of the chamber in Panc05.04 cells transfected with sh-NUDT21 plasmid. **G**, **H** Wound healing assay shows the wound healing rate in Panc05.04 cells transfected with OE-NUDT21 plasmid. **I** Cell colony formation assay shows the colony size in Panc05.04 cells transfected with sh-NUDT21 and OE-NUDT21 plasmid. **J**, **K** The ATP concentration in the culture media was measured in Panc05.04 cells transfected with sh-NUDT21 and OE-NUDT21 plasmid. **L**, **M** NADPH / NADP^+^ ratios in the culture media were measured in Panc05.04 cells transfected with sh-NUDT21 and OE-NUDT21 plasmid

Wound healing assay and transwell migration assay were used to further investigate the role of NUDFS2 in the migration of pancreatic cell lines. The result showed that the wound healing rate of Panc05.04 cells transfected with sh-NUDT21 plasmid was lower compare to the control group (Fig. 4A, B), whereas it was higher compared to the control group when transfected with OE-NUDT21 plasmid (Fig. 4C, D). Transwell migration assay showed a number of transitional cells that migrated to the bottom of the chamber in Panc05.04 cells are less compared to normal control when transfected with sh-NUDT21 plasmid (Fig. 4E, F), while the cells transfected with OE-NUDT21 plasmid are more compared to normal control (Fig. 4G, H). The results of colony formation assay also showed that the colony size of Panc05.04 cells transfected with sh-NUDT21 plasmid was smaller compared to control group, while it was larger compared to control group when transfected with OE-NUDT21 plasmid (Fig. 4I). To further validate the effects of NUDT21 on cellular redox function and energy metabolism, the NADPH/NADP^+^ ratio and ATP concentration were also measured in the culture media. The result showed that the NADPH/NADP^+^ ratio and ATP concentration in Panc05.04 cells transfected with sh-NUDT21 plasmid were lower compared to control group, while the content in Panc05.04 cells transfected with OE-NUDT21 plasmid was higher compared to control group (Fig. 4J-M).

### Identification of NUDT21 interactors by mass spectrometry

**Fig. 5:**
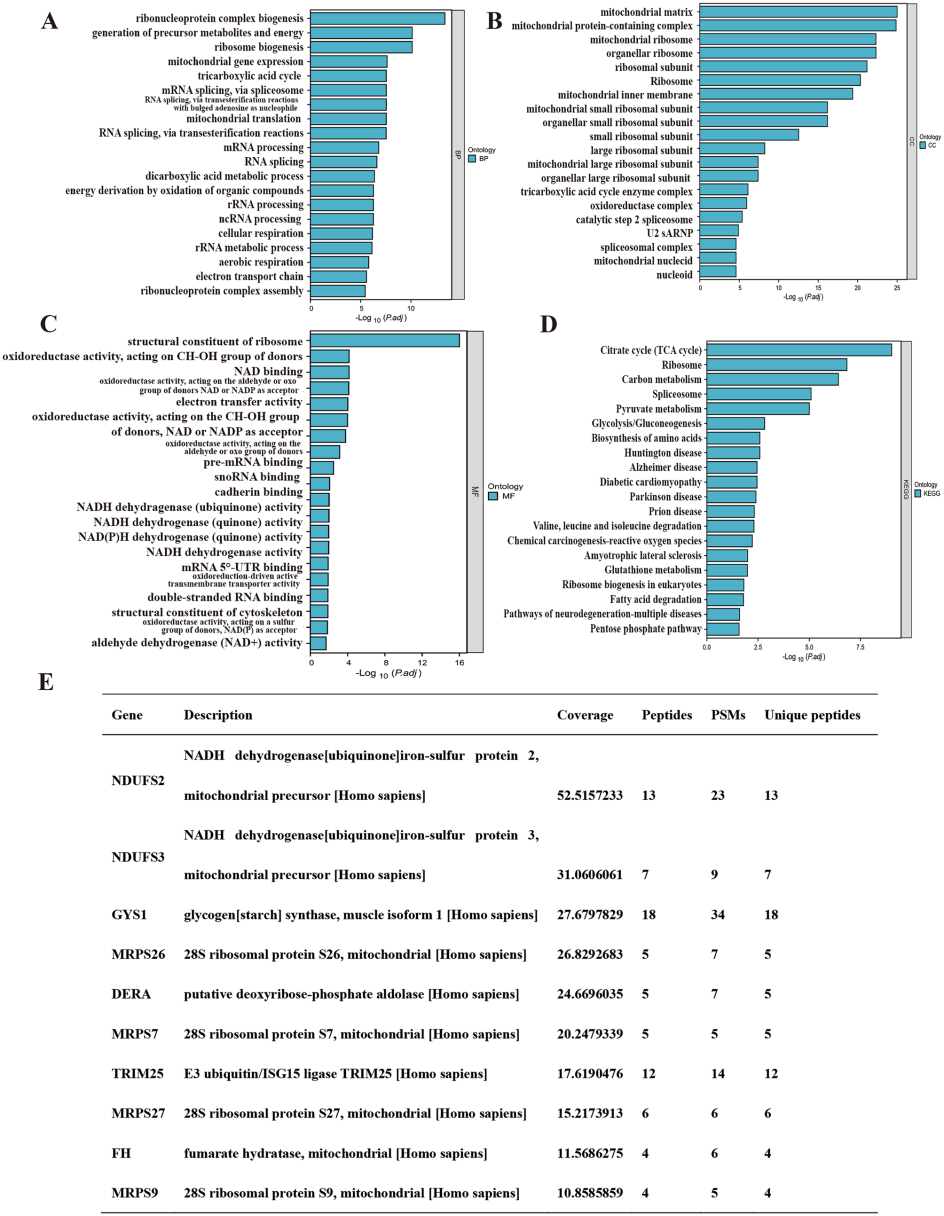
Identification of NDUFS2-interacting proteins via mass spectrometry. **A**–**D** A total of 17,726 NUDFS2-interacting proteins were identified by mass spectrometry. Enriched analysis of GO and KEGG pathway were shown. **E** A list of candidate NDUFS2-interacting proteins for further validation

We overexpressed NUDT21 through transduction of OE-NUDT21 plasmid and carried out co-immunoprecipitation (co-IP) to capture NUDT21 interactors. We identified 17,726 potential interactors using mass spectrometry. Next, we subjected these candidate interactors to GO and KEGG pathway analysis using DAVID and identified the top 20 terms regulated by NUDT21 in biological process (BP), cellular component (CC), molecular function (MF), as well as KEGG pathway as shown in Fig. 5A–D. These results indicate that potential NUDT21 interactions are tightly associated with mitochondrial function. Based on the number of unique peptides, the top 10 target genes were selected for further experimental validation (Fig. 5E).

### NUDT21 interacted with NDUFS2 in pancreatic cancer

**Fig. 6:**
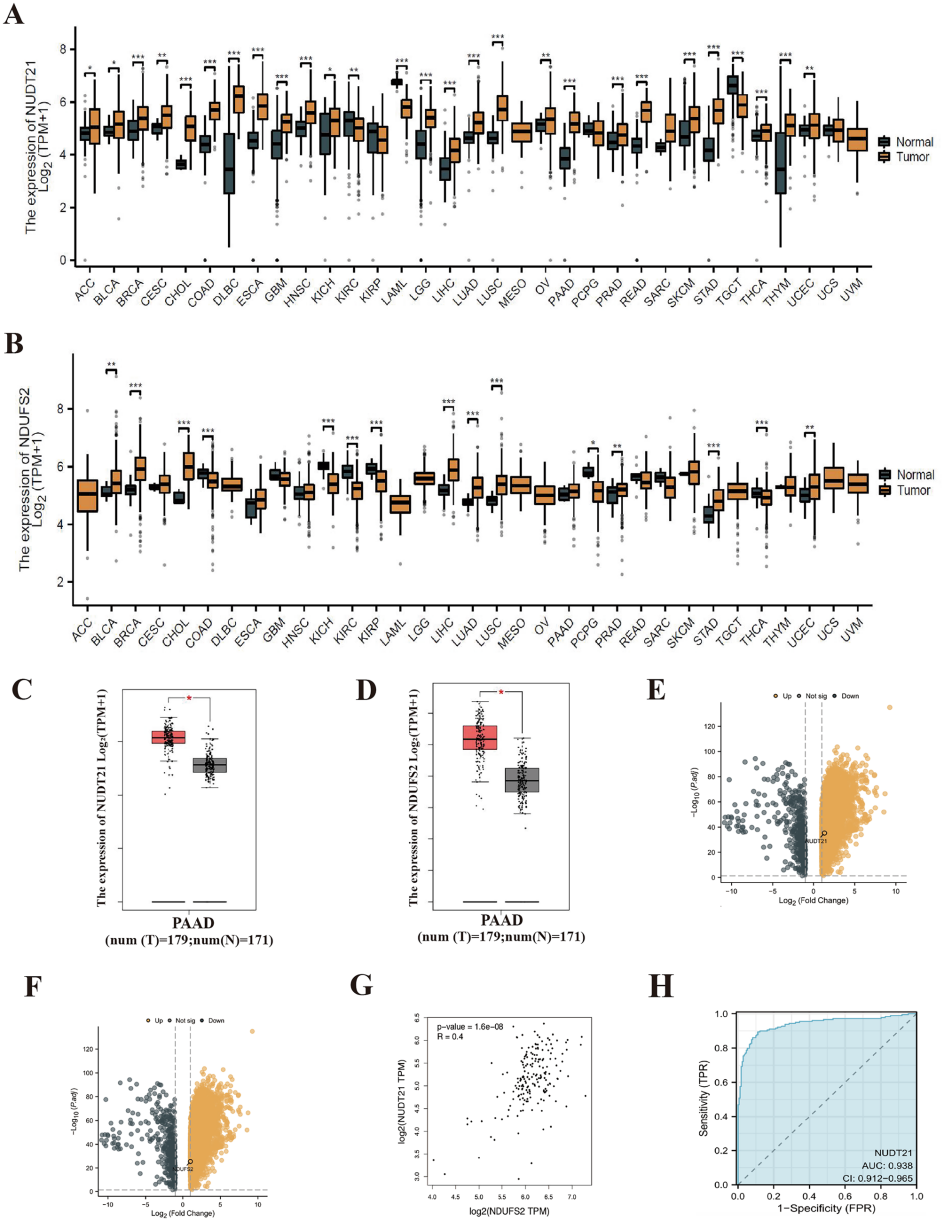
Differential expression of NUDT21 in PAAD and normal tissues. **A** Results of differential analysis of NUDT21 expression in 33 tumors based on the data in the TCGA database. **B** Results of differential analysis of NDUFS2 expression in 33 tumors based on the data in the TCGA database. **C** Expression of NUDT21 at transcriptional level in PAAD and normal tissues. **D** Expression of NDUFS2 at transcriptional level in PAAD and normal tissues. **E** Volcano plot of single-gene differential analysis of NUDT21. **F** Volcano plot of single-gene differential analysis of NDUFS2. **G** The correlation between NDUFS2 and NUDT21 was analyzed according to the TCGA database online (R = 0.4, *P* < 0.01). **H** ROC curve, the area under the ROC curve is between 0.5 and 1. The closer the AUC is to 1, the better the diagnosis is. The AUC is between 0.5 and 0.7 with low accuracy, the AUC is between 0.7 and 0.9 with some accuracy, and the AUC is above 0.9 with high accuracy

To find the downstream regulatory molecules of NUDT21, we also screened the TCGA database and looked for molecules that could also play an important role in mitochondria according to the result of mass spectrometry and we discovered the NDUFS2. We screened the expression of NDUFS2 in major human cancer species using the TCGA database and GTEx data, and we found that NUDT21 and NDUFS2 were both significantly higher expressed in pancreatic adenocarcinoma (PAAD) compared to control tissues (Fig. 6A–D). These results demonstrated that NUDT21 had tissue specificity in different kinds of human cancers. We also filtered all dysregulated genes in pancreatic cancer profiles from TCGA database, and we identified all the upregulated genes and downregulated genes; the Volcano map deciphers the localization of NUDT21 and NDUFS2 in PAAD among all the upregulated genes (Fig. 6E, F). We analyzed the correlation between NUDT21 and NDUFS2 via GEPIA online database, and the result showed that NUDT21 was positively correlated with NDUFS2 (R = 0.4; *P* < 0.01; Fig. 6G). We constructed a ROC curve to verify the accuracy of NUDT21 expression in predicting prognosis, and the results are shown in Fig. 6H. The predictive ability of the NUDT21 was relative accurate in predicting the diagnostic outcome of tumor patients versus normal patients (AUC = 0.938, CI = 0.912–0.965).

### Verification of NUDT21-interacting proteins using co-immunoprecipitation

**Fig. 7:**
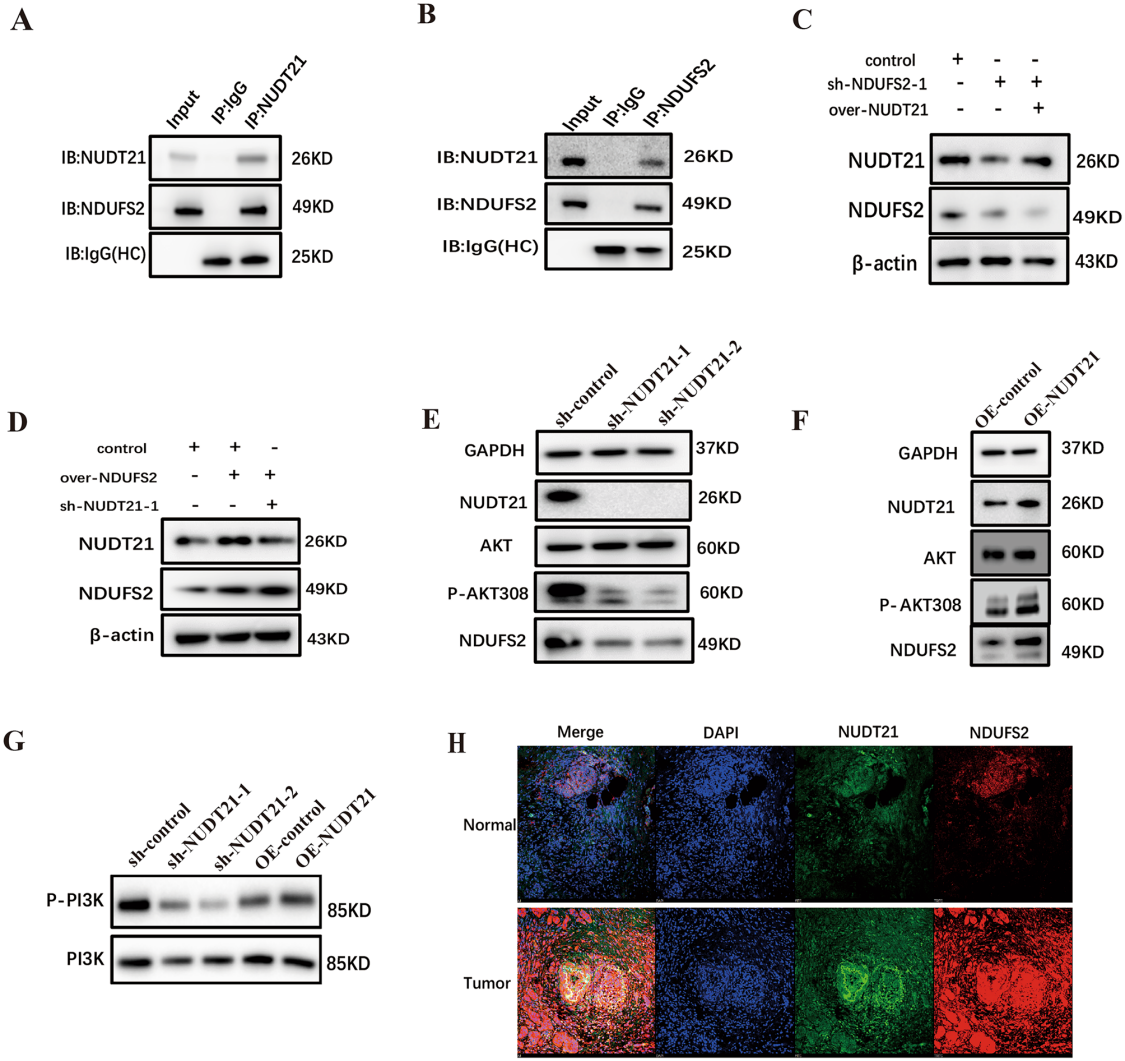
NUDT21 activates PI3K–AKT pathway in pancreatic cancer. **A** NUDT21 interacted with NDUFS2 were found via immunoprecipitation assay. **B** NDUFS2 interacted with NUDT21 were found via immunoprecipitation assay. **C** Western blotting detecting the expression of NUDT21 in Panc05.04 cells when co-transfected with sh-NDUFS2 and OE-NUDT21plasmid. **D** Western blotting detecting the expression of NUDT21 when co-transfected Panc05.04 cells with OE-NDUFS2 and sh-NUDT21 plasmid. **E**, **F** Western blotting detecting the expression of NDUFS2, P-AKT308 when transfected with sh-NUDT21 and OE-NUDT21 plasmid in Panc05.04 cells. **G** Western blotting detecting the expression of P-PI3K when transfected with sh-NUDT21 and OE-NUDT21 plasmid in Panc05.04 cells. **H** Confocal analysis shows the expression of NUDT21 and NDUFS2 in pancreatic cancer and normal control (Scale bar: 10 μm; Magnification: 100 ×)

Using bioinformatics analysis of proteins most likely to interact with NUDT21, we selected NDUFS2 for validation. We performed Western blotting to detect the presence of NDUFS2 following co-IP. Interaction between NUDT21 and NDUFS2 was analyzed by immunoprecipitation with IgG or an anti-NUDT21 antibody in Panc05.04 cells (Fig. 7A). Next, we confirmed the interaction between NUDT21 and NDUFS2 by reciprocal co-IP (Fig. 7B). To examine whether the promoting role of NUDT21 in pancreatic cancer cells was mediated by NDUFS2, rescue experiments were performed in PANC05.04 cells. As shown in Fig. 7C, the protein levels of NUDFS2 decreased significantly after transfected with sh-NDUFS2-1, but these decreases were abolished by co-transfection with NUDT21 overexpressing plasmids (OE-NUDT21). Meanwhile, the protein levels of NUDFS2 were overexpressed significantly after transfected with OE-NDUFS2 plasmid, but the overexpression were abolished by co-transfection with sh-NUDT21 plasmids (Fig. 7D).

To further search for the downstream signaling pathways through which NUDT21 promotes pancreatic cancer cell proliferation and migration by interacting with NDUFS2, we validated the PI3K–AKT pathway postulated by GO and KEGG analysis. Western blotting showed that the levels of NDUFS2 and P-AKT308 were dramatically downregulated (Fig. 7E), while caspase3 was upregulated in NUDT21-silenced samples (Fig. S1A). After overexpression of NUDT21 in Panc05.04 cells, we found that the expression of NDUFS2 and P-AKT308 was dramatically upregulated (Fig. 7F), while caspase3 was downregulated (Fig. S1B). We hypothesize that NUDT21 may be the upstream regulatory molecule for NDUFS2. We also found that P-PI3K expression was significantly downregulated after interfering with NUDT21 and significantly upregulated after overexpressing NUDT21 (Fig. 7G). We also examined the changes of P-AKT308 after interfering with NDUFS2 at the same time, and we found that the expression of P-AKT308 was highest after 5 min of treatment with the P-AKT activator, insulin (Fig. S1C), and the expression of P-AKT308 was further elevated when interfering NDUFS2, suggesting that interfering with NDUFS2 may promote the phosphorylation of AKT308 (Fig. S1D). To further validate the interaction between NUDT21 and NDUFS2, IF assay was performed on pancreatic cancer and adjacent tissues obtained from surgery, and the results showed that NUDT21 and NDUFS2 were co-localized in pancreatic cancer and they were both highly expressed in pancreatic cancer than adjacent tissues (Fig. 7H).

## Discussion

Currently, Multi-disciplinary Treatment (MDT), including surgery and chemotherapy, is considered the gold-standard treatment for pancreatic cancer. Neoadjuvant chemotherapy, genetically engineered modified T-cell therapy, CAR-T cells, autophagy, MicroRNAs, tumor vaccines, and targeted therapies have provided new approaches for the treatment of pancreatic cancer (Gugenheim et al. 2022; Leidner et al. 2022; Sadelain 2015; Wang et al. 2021; Chu et al. 2021; Moore et al. 2007), but the outcomes were not always satisfactory. Hence, we urgently need to find more therapeutic targets to provide more treatment options for pancreatic cancer.

NUDT21, a 3′ end processing complex conserved in multicellular organisms but absent from yeast (Darmon and Lutz 2012), is one of the main regulators of 3′ UTR(3′ untranslated regions) length (Li et al. 2015). NUDT21 plays an important physiological function in the occurrence and development of many diseases (Tellier et al. 2018). In this study, the expression profiles of NUDT21 in multiple cancers were analyzed based on TCGA and GEO databases. Then, NUDT21 expression in pancreatic cancer was investigated by gene differential analysis and single-gene correlation analysis. In addition, we performed GO and KEGG pathway analysis, gene enrichment analysis, and Kaplan–Meier survival analysis to predict the potential function of NUDT21 and its role in the prognosis of pancreatic cancer patients. We found that patients with high expression of NUDT21 showed poor OS rates compared with patients with low expression of NUDT21.

The correlation between NUDT21 expression and 24 types of immune cells in PAAD showed that NUDT21 expression was negatively correlated with NK cells, pDC, and Th17 cells. Immune cell infiltration improves poor patient prognosis, whereas low concentrations of infiltrating immune cells can lead to immune escape from cancer cells, resulting in poor prognosis (Buisseret et al. 2018; Chen et al. 2020). In addition, the results of immune infiltration showed that the degree of Th2 cell infiltration was significantly positively correlated with the expression of NUDT21. The transition from Th1/Th2 balance to Th2 dominance is a crucial factor in tumor progression, and Th2 cells are detrimental to the antitumor effects of cellular immunity. Restoring the balance between Th1 and Th2 cells is of great significance in the treatment of tumors (Sharma et al. 2007; Johnson et al. 2014). We also found that most of the immune checkpoints currently used in therapy (e.g., PDCD1, CTLA4) were positively correlated with the expression of NUDT21, implying that it may be possible that PAAD may benefit more from immune-associated therapies against these checkpoints. All these results indicate that upregulation of NUDT21 expression can inhibit the antitumor immune response in PAAD patients.

Apart from the predictions of the bioinformatic data, we further verified the conclusions in vitro. We systematically examined the functional role of NUDT21 in human pancreatic cancer cells. shRNA-mediated depletion of NUDT21 dramatically decreased cell viability, cell colony formation, cell proliferation, cell migration in pancreatic cancer cells as determined by cell apoptosis assay, cell colony formation assay, and cell migration assay, respectively. Overexpression of NUDT21 concordantly promoted both cell proliferation and migration in pancreatic cancer cells. Moreover, we found that NUDT21 regulates pancreatic cancer cell proliferation and migration by modulating and stabilizing NDUFS2 and activating the PI3K-AKT signaling pathway through protein mass spectrometry. Compared with normal pancreatic tissues, NUDT21 was expressed at a higher level in human pancreatic cancer tissues, which was consistent with the prediction of the bioinformatic results.

The phosphoinositide 3-kinase (PI3K)–AKT pathway is the most commonly activated pathway in human cancers (Lawrence et al. 2014). AKT-mediated phosphorylation of these protein targets serves to influence a variety of cell biological functions, including cell growth, proliferation, survival and metabolism (Manning and Toker 2017). Many anticancer therapies including cytotoxic drugs, radiotherapy, or immunotherapy can cause tumor cell death by activating caspase3 (Zhou et al. 2018). AKT/mTOR signaling is also a major determinant of cellular energy metabolism (Manning and Toker 2017; Saxton and Sabatini 2017). After interfering with NUDT21, the PI3K–AKT pathway was inhibited and P-AKT308 expression was downregulated, while after overexpression of NUDT21, the PI3K–AKT pathway was activated and P-AKT308 expression was upregulated. Phosphorylated AKT308 inhibited the expression of caspase3, which promoted the proliferation and migration of pancreatic cancer cells and inhibited apoptosis.

NDUFS2 (NADH: ubiquinone oxidoreductase core subunit S2) is localized in mitochondria and is the core subunit of the mitochondrial respiratory chain complex I, which is encoded by nuclear DNA. It has been shown that after interfering with the intracellular NDUFS2, the activity of Complex I is inhibited, intracellular ATP production is reduced, tumor growth is suppressed, and tumor metastasis is significantly reduced (Liu et al. 2019). In our study, we found that interfering with NUDT21 also inhibited intracellular ATP synthesis and decreased the NADPH/NADP^+^ ratio, which means that NDUFS2 could play an important role in the ATP synthesis and redox system.

In conclusion, our results showed that NUDT21 was highly expressed in pancreatic cancer tissues compared with paraneoplastic tissues, and the higher the expression, the worse the prognosis of the patients. NUDT21 promotes the proliferation and migration of pancreatic cancer cells through the regulation and stabilization of NDUFS2 and activation of the PI3K–AKT pathway. NUDT21 can be used as a diagnostic and prognostic marker for pancreatic cancer patients in clinic.

## Supplementary Information

Below is the link to the electronic supplementary material.

Supplementary file1 (TIF 8307 KB) Figure S1 (A,B)Western blotting detecting the expression of caspase8 when transfected with sh-NUDT21 and OE-NUDT21 plasmid in Panc05.04 cells. (C)Western blotting detecting the expression of P-AKT308 at different point of time. (D) Western blotting detecting the expression of P-AKT308 transfected with sh-NDUFS2 plasmid and treated with insulin or wortmannin for 5 mins

## Data Availability

The datasets generated during and/or analyzed during the current study are available from the corresponding author on reasonable request.
