# Supplementary figures and images for: NUDT21 interacts with NDUFS2 to activate the PI3K/AKT pathway and promotes pancreatic cancer pathogenesis
Source: J Cancer Res Clin Oncol. 2024 Jan 9;150(1):8. doi: 10.1007/s00432-023-05540-1 (PMC10776698; doi:10.1007/s00432-023-05540-1)

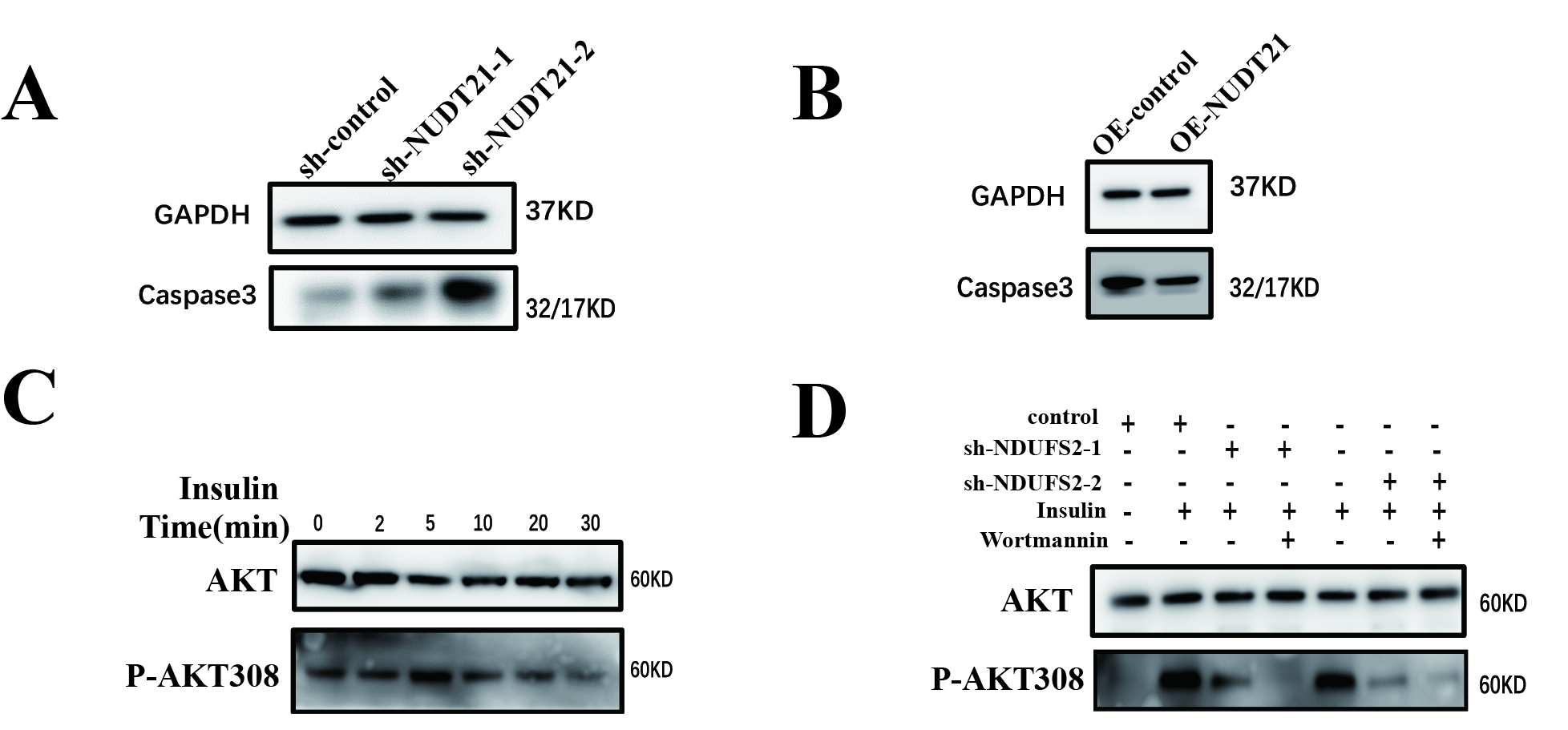

Supplement: Supplementary file 1 — Supplementary file1 (TIF 8307 KB) Figure S1 (A,B)Western blotting detecting the expression of caspase8 when transfected with sh-NUDT21 and OE-NUDT21 plasmid in Panc05.04 cells. (C)Western blotting detecting the expression of P-AKT308 at different point of time. (D) Western blotting detecting the expression of P-AKT308 transfected with sh-NDUFS2 plasmid and treated with insulin or wortmannin for 5 mins [file 432_2023_5540_MOESM1_ESM.tif]
